# Comparative evaluation of a novel solar powered low-cost ophthalmoscope (Arclight) by eye healthcare workers in Malawi

**DOI:** 10.1136/bmjinnov-2017-000225

**Published:** 2018-02-12

**Authors:** Rebecca Blundell, David Roberts, Evridiki Fioratou, Carl Abraham, Joseph Msosa, Tamara Chirambo, Andrew Blaikie

**Affiliations:** 1 School of Medicine, University of Dundee, Dundee, UK; 2 Department of Ophthalmology, Ninewells Hospital, Dundee, UK; 3 Department of Optometry, Malawi College of Health Sciences, Lilongwe, Malawi; 4 Lions Sight First Eye Unit, Kamuzu Central Hospital, Lilongwe, Central Region, Malawi; 5 Eye Department, Nkhoma Hospital, Nkhoma, Malawi; 6 Global Health Implementation, School of Medicine, University of St Andrews, St Andrews, UK

**Keywords:** ophthalmoscopes, blindness, diabetic retinopathy, optic nerve diseases, culturally appropriate technology

## Abstract

This study compared a novel low-cost solar powered direct ophthalmoscope called the Arclight with a traditional direct ophthalmoscope (TDO). After appropriate training, 25 Malawian eye healthcare workers were asked to examine 12 retinal images placed in a teaching manikin head with both the Arclight ophthalmoscope and a traditional direct ophthalmoscope (Keeler Professional V.2.8). Participants were scored on their ability to identify clinical signs, to make a diagnosis and how long they took to make a diagnosis. They were also asked to score each ophthalmoscope for ‘ease of use’. Statistically significant differences were found in favour of the Arclight in the number of clinical signs identified, correct diagnoses made and ease of use. The ophthalmoscopes were equally effective as a screening tool for diabetic retinopathy, and there was no statistically difference in time to diagnosis. The authors conclude that the Arclight offers an easy to use, low cost alternative to the traditional direct ophthalmoscope to meet the demands for screening and diagnosis of visually impairing eye disorders in low-income and middle-income countries.

## Introduction

Over 285 million people worldwide suffer from significant visual impairment of which approximately 60%–70% is considered to be preventable and treatable.^1 2^ The majority of cases reside in low-income and middle-income countries (LMICs)^1^ where access to diagnostic tools is least.^3 4^ Early identification and treatment, particularly of those conditions affecting the retina and optic nerve, relies on tools such as the traditional direct ophthalmoscope (TDO).

Although the TDO has traditionally been one of the main ophthalmic screening and diagnostic tools, it has limitations of high initial cost of purchase, requirement for regular maintenance (bulbs and batteries) and perceived difficulty of use.^5 6^ Consequently, the ‘functional’ availability of this important device in LMICs is limited.

The Vision 2020 Right to Sight initiative is a global strategy, launched in 1999, with the aim to eliminate avoidable blindness by 2020.^7^ The initiative has three main aims: disease control, human resource development and infrastructure strengthening including relevant technology development for eye care delivery.^8^ Along with the Lancet Commission,^9^ recommendations have emphasised the development of ‘frugal’ and culturally appropriate technology for users in LMICs.

**Figure 1:**
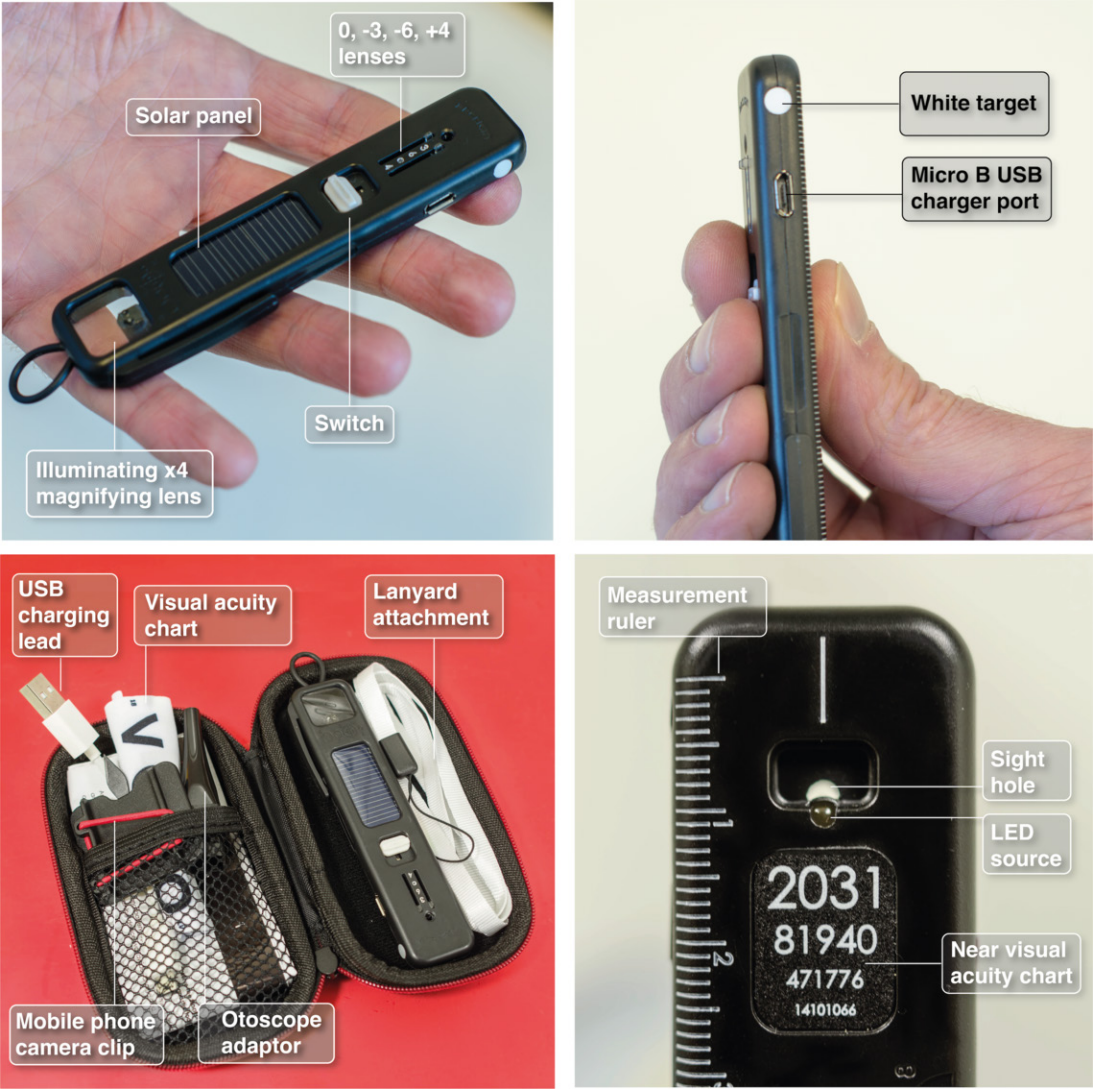
The Arclight with selected features highlighted.

In response to these challenges, a novel low-cost multipurpose diagnostic device has been developed by Arclight Medical^10^ employing a number of innovative and unique design features (figure 1).

**Figure 2:**
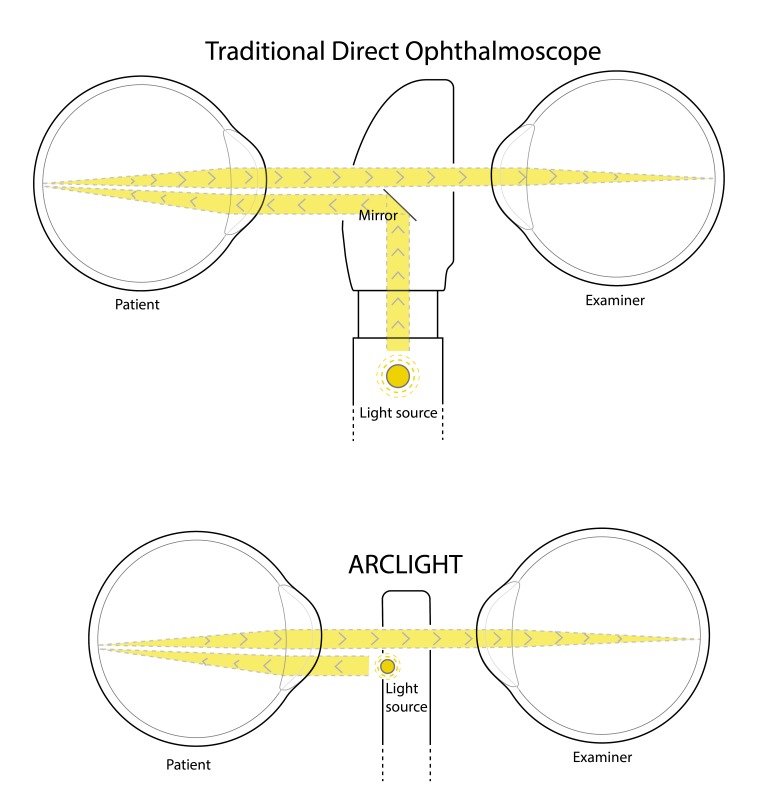
The Arclight illumination uses a light emitting diode placed directly below the viewing hole, facing the patient, creating a near-axial light source unlike the traditional direct ophthalmoscope, which uses a mirror to redirect light into an axial path.

The key patented feature of the device is to employ a Light Emitting Diode (LED), which can be charged by an integrated photovoltaic (solar) panel. This novel design eliminates the need for bulky, hard to source and expensive replacement filament bulbs and batteries and mains electricity charging. The LED is small enough to be placed directly below the viewing hole, facing the patient, creating a near axial light source (figure 2), avoiding using a mirror to redirect light from below as is traditionally done. These design changes create a slim (110 mm long×26 mm wide×9 mm thick) and light device (18 g).

Additionally, the Arclight has an integrated magnifying loupe and otoscope broadening its diagnostic potential. As it is available at only £5.00 to low-income users when sold in bulk,^11^ it is potentially an economically and practical alternative to TDOs in LMICs. As yet, however, no studies have evaluated the effectiveness of the Arclight in diagnosing retinal disorders (including diabetic retinopathy) among eye care providers in a LMIC.

### The aim of this study

The aim of the study is to compare how the Arclight device performs in comparison to a TDO when used by Malawian eye healthcare professionals examining the fundi of simulated eyes.

## Materials and methods

Appropriate ethical approval was obtained. Twenty-five eye healthcare professionals and optometry students were recruited with fully informed consent. Participants were recruited from two hospital eye departments and one school of optometry.

The participants were already familiar with using a TDO. An introductory ‘refresher’ session was provided on direct ophthalmoscopy and the examiners had a brief training session with the Arclight to familiarise themselves with the practicalities of how the device works.

### Clinical signs, diagnosis and ease of use (EOU) scores

**Figure 3:**
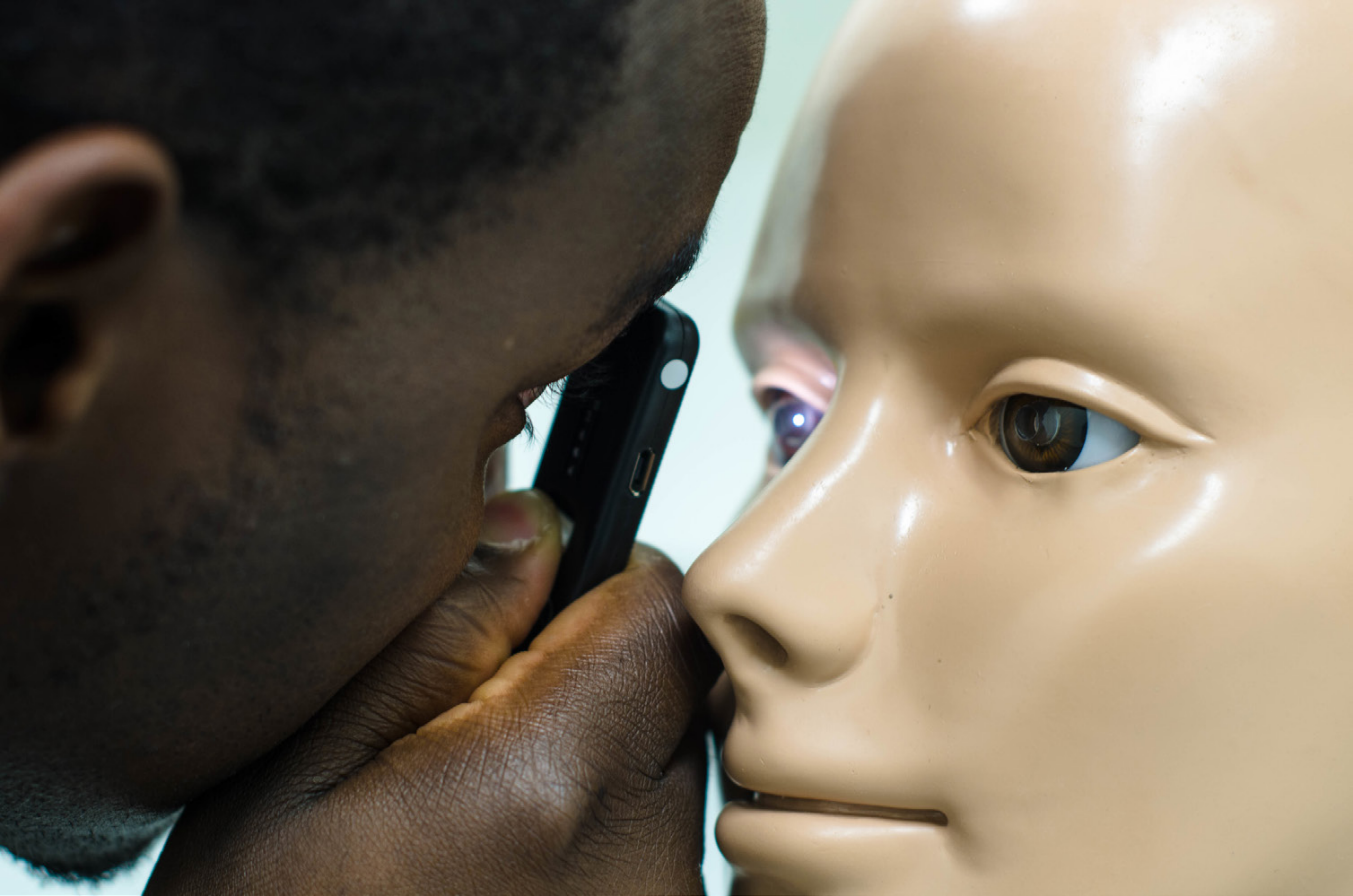
The Arclight being used with teaching slides in a manikin head.

Participants were asked to examine 12 retinal pathology slides placed in an Adam-Rouilly teaching manikin head (figure 3). Slides included background retinopathy and diabetic maculopathy, preproliferative retinopathy, advanced proliferative retinopathy, diabetic maculopathy, pan laser photocoagulation, normal fundus, glaucomatous disc, papilloedema/swollen disc, toxoplasmosis scar/optic atrophy, cytomegalovirus, central retinal vein occlusion and central retinal artery occlusion. A cross-over design was utilised with 12 participants using the Arclight first (group 1) and the TDO second and 13 participants the converse (group 2). The TDO used in this study was a Keeler Professional V.2.8. The order of the slides was randomised before ‘crossing over’.

The participants were asked to describe the clinical signs they saw on the slide and to provide a diagnosis. Every clinical sign correctly identified received one mark. The time taken to reach a diagnosis was also recorded. Correct diagnoses, recognition of clinical signs and time taken to diagnose were normally distributed and a paired t-test was used to assess statistical significance.

Participants were additionally asked to rate the EOU as described below:

1. Could not use this ophthalmoscope to see the red reflex.
2. Could see the red reflex.
3. Could not focus the fundus.
4. Could see vessels but not optic disc.
5. Could see the disc and retinal fields but it was not in focus.
6. Could see the disc and retinal fields in focus with high level of difficulty.
7. Could see disc and retinal fields in focus with medium level of difficulty.
8. Could see disc and retinal fields in focus with low level of difficulty.

For descriptive statistics, an EOU score greater than 7 was defined as ‘easy’ and less than 6 (</6) as ‘difficult’. The EOU score frequency was not normally distributed and a non-parametric Wilcoxon sign rank test was applied.

## Results

### Identification of clinical signs

A possible 53 pathological features could be diagnosed in the 12 slides. Arclight mean score per participant was 31.6 (95% CI 28.85 to 34.35) and TDO 28.72 (95% CI 26.42 to 31.01). Participants using the Arclight were significantly better at correctly identifying clinical signs compared with the TDO; the mean difference in the total number of correctly identified features by each participant was 2.89 in favour of the Arclight, t=3.285, P=0.003, 95% CI 1.13 to 4.65.

### Pathology diagnosis

Fourteen possible diagnoses could be made from the 12 slides; Arclight mean score was 9.8 (95% CI 8.87 to 10.73) and the TDO 9.12 (95% CI 8.23 to 10.01). Users of the Arclight were significantly better at correctly diagnosing the pathology illustrated on the slide; the mean difference in the number of slides correctly diagnosed by participants was 0.685 in favour of the Arclight, t=2.957, P=0.007, 95% CI 0.21 to 1.17.

Four of the 12 slides displayed solely signs of diabetic retinopathy (DR), so a maximum of 100 DR slides could be identified by all 25 participants, using the Arclight; participants correctly identified 83 slides with DR signs and the TDO 79 slides. When analysing theses signs, the sensitivity of the Arclight was 83% (95% CI 75.63 to 90.36) and TDO 79% (95% CI 71.01 to 87.00) and specificity of the Arclight was 98% (95% CI 96.06 to 99.94) and TDO 97.5% (95% CI 95.34 to 99.66) demonstrating that the devices were comparable to each other as a screening tool.

### Time to diagnosis

Arclight mean time per slide was 93.19 s (95% CI 80.40 to 105.98) and TDO mean 103.15 s (95% CI 85.99 to 120.31). There was a non-significant trend for participants to make faster diagnoses with the Arclight; the mean difference between the time taken by participants to diagnose each slide was −8.95 s in favour of the Arclight, t=1.978, with P=0.06, 95% CI −18 to 0.1.

### EOU score

For every examination, the user recorded a subjective EOU score; overall 300 examinations were performed with each device. More examinations were rated as ‘easy’ (defined as a score greater than 7) when using the Arclight (68%) compared with the TDO (59%). Mean score for Arclight was 6.7 (SEM±0.16) and TDO 6.5 (SEM±0.16). Using the Wilcoxon Sign rank test, this was statistically significant (P=0.01).

## Discussion

Health clinics in LMICs often become ‘grave yards’ of redundant non-functional devices.^9^ Diagnostic tools are usually designed for health systems of wealthy countries, leading to expensive and overly complex devices, which are unnecessarily challenging to use and hard to maintain.^3^

This study has shown that the Arclight can satisfy the recommendations of the Vision 2020^12^ initiative and the Lancet Commission^9^ to develop devices for LMICs that are low cost, portable and independent of scarce and expensive consumables while not compromising the core function. In the hands of Malawian eye care workers, the Arclight, despite being many fold less expensive than TDOs, performed better. This supports Lowe *et al*’s^5^ previous study demonstrating the Arclight to be a suitable alternative to the TDO for the purpose of screening and accurately diagnosing optic nerve disease.

Our study is also consistent with this previous study demonstrating the Arclight to be easier to use than a TDO. Possible reasons for this include the simplified design of the Arclight with a minimum of switches and dials creating an intuitive and uncomplicated device. Also, as previously mentioned, the Arclight is small, light and importantly very slim (9 mm thick) allowing the user to get closer to the pupil plane of the patient potentially offering a wider more stable field of view (figure 2). These features may also help to explain why users are more accurate at identifying retinal signs and make more correct diagnoses compared with when using a TDO.

There were, however, important limitations to this study. An Adam Rouilly teaching head with simulated eyes cannot replicate media opacity, variability in pupil size or the head and eye movement of a real patient and consequently further comparative studies using real patients and in particular those with diabetic retinopathy should be considered in the future.^5 13^

An additional limitation is that participant’s previous training, experience and familiarity with a TDO may have negatively influenced their impression of the Arclight.^6^ Despite this, the Arclight was considered easier to use than the TDO in this study emphasising the simplified and intuitive design as a major strength of the device.

Further studies should explore the other functions of the device, including the anterior segment loupe, which allows differentiation of causes of medial opacity (eg, cataract vs corneal scar) and a magnified view of the tarsal plates for diagnosis and grading trachoma.^14^ Additionally, no studies have evaluated the effectiveness of the otoscope function.

## Conclusion

In conclusion, this study provides additional evidence that the Arclight is a genuine practical and economic alternative to the TDO. By overcoming barriers to acquisition, maintenance and practical use that are present in low-resource settings, the device represents an important addition to the current strategies being employed to reduce the global burden of blindness.^2^ In addition, the Arclight can also function as a loupe and otoscope and consequently could have a broader role to play in healthcare.
